# Supplementary material for: The Divider Assay is a high-throughput pipeline for aggression analysis in Drosophila
Source: Commun Biol. 2021 Jan 19;4:85. doi: 10.1038/s42003-020-01617-6 (PMC7815768; doi:10.1038/s42003-020-01617-6)
Supplement: Supplementary file 2 — Supplementary Information [file 42003_2020_1617_MOESM2_ESM.pdf]

Supplementary Information

The Divider Assay is a high-throughput pipeline for aggression analysis in *Drosophila*.

Budhaditya Chowdhury[1], Meng Wang[1], Joshua P. Gnerer[1], and Herman A. Dierick[1,2]

Supplementary Figure 1:

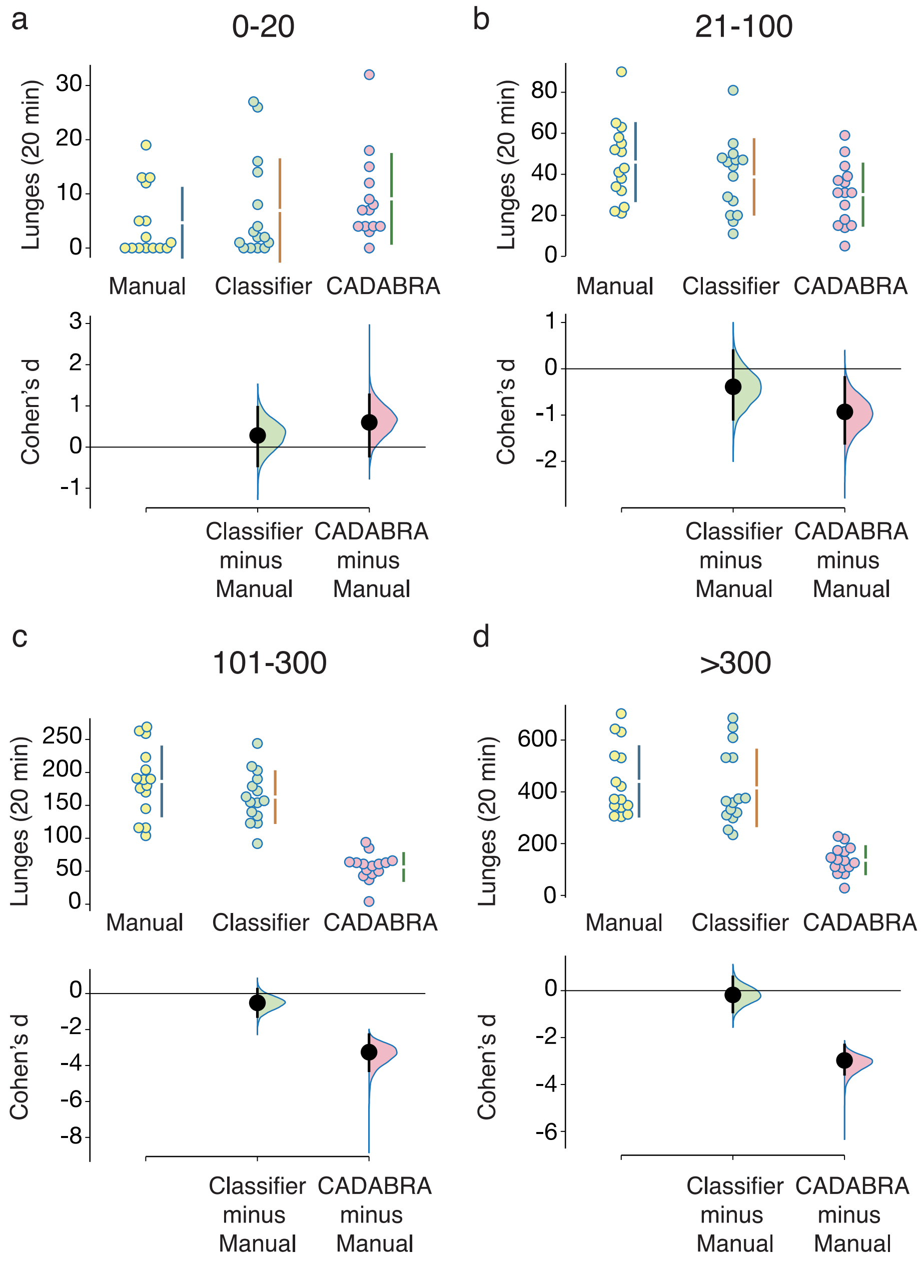

Quantifying CADABRA error. To better visualize the error in lunge number quantification of the CADABRA software, we also plotted Cohen's d for the Classifier and CADABRA comparisons against the gold standard of Manual scoring using a publicly available web application (for more details see Methods). The upper plots show the raw data as in Fig. 1C and the lower plots show the mean differences for the different fighting intensity groups (0-20, 21-100, 101-300, and >300). The Cohen's d values are shown as dots with the 95% confidence intervals from 5,000 bootstrap samples. **a.** For **0-20** lunges: Cohen's d is 0.282 for manual versus classifier [95.0%CI -0.458, 0.968, p = 0.39], and 0.60 for manual versus CADABRA [95.0%CI -0.219, 1.27, p = 0.067]. **b.** For **21-100** lunges: Cohen's d is -0.388 for manual versus classifier [95.0%CI -1.09, 0.394, p = 0.254], and -0.93 for manual versus CADABRA [95.0%CI -1.61, -0.185, p = 0.021]. **c.** For **101-300** lunges: Cohen's d is -0.516 for manual versus classifier [95.0%CI -1.28, 0.243, p = 0.191], and -3.26 for manual versus CADABRA [95.0%CI -4.29, -2.29, p = 3.37e-06]. **d.** For **>300** lunges: Cohen's d is -0.179 for manual versus classifier [95.0%CI -0.908, 0.592, p = 0.561], -2.97 for manual versus CADABRA [95.0%CI -3.56, -2.31, p = 3.39e-06].

Supplementary Figure 2:

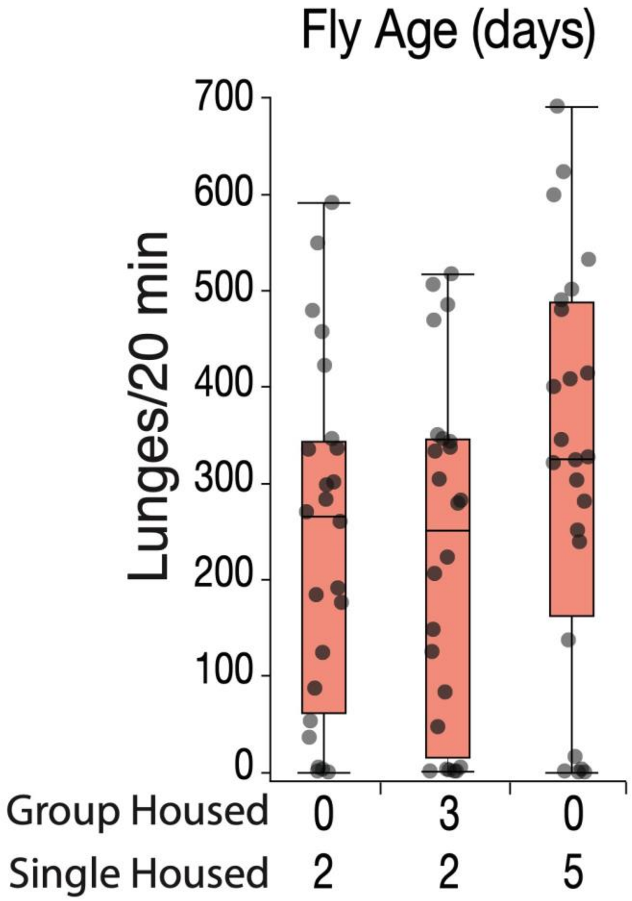

Isolation time affects aggression. Flies isolated and aged for 2d have almost identical lunge numbers as 5d old flies isolated for 2d and have lower lunge numbers than 5d old flies isolated for 5d (Kruskal-Wallis ANOVA, $p = 0.29$, n = 24 pairs per group). Boxplots show the median, 1st and 3rd quartiles as boxes, with whiskers representing the 5 and 95% intervals. Boxplots show the median, 1st and 3rd quartiles as boxes, with whiskers representing the 5 and 95% intervals.

Supplementary Figure 3:

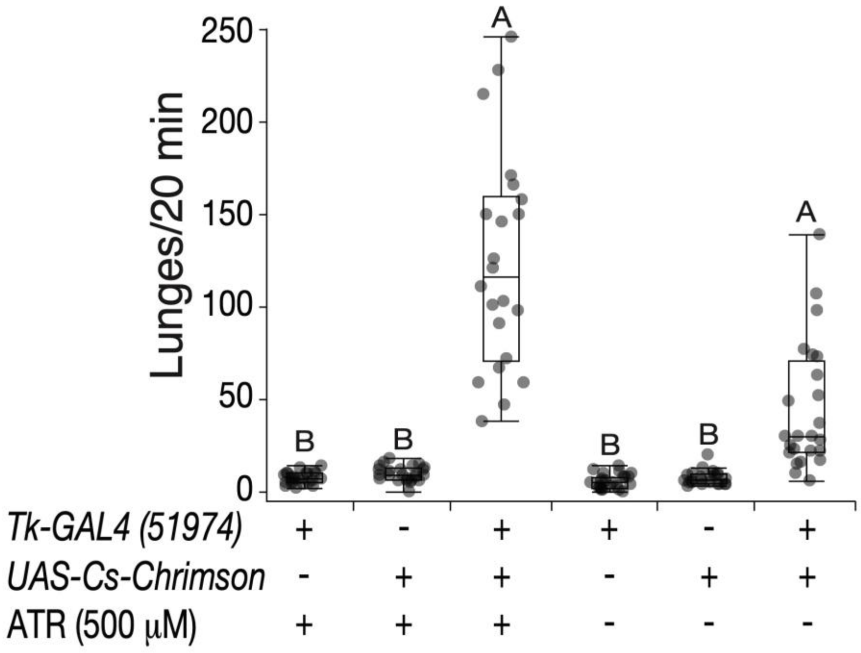

Lunge analysis on existing aggression lines. To evaluate performance of our Divider Assay on a strain previously reported to have increased aggression[49], we tested *Tk-GAL4* flies expressing the red shifted optogenetic effector, *UAS-CsChrimson*. Flies with an activated *Tk-GAL4* circuit showed a strong increase in lunge numbers compared to controls. Even in the absence of the Vitamin A chromophore ATR (All-Trans-Retinal) the *Tk-GAL4>UAS-CsChrimson* flies respond to light exposure, although the effect is much less pronounced than the ATR treated group (see Methods for details) (Kruskal Wallis ANOVA with Dunn's test and Bonferroni correction, statistically significantly different groups are denoted with a different letter, n = 22-24 pairs per group). Boxplots show the median, 1st and 3rd quartiles as boxes, with whiskers representing the 5 and 95% intervals. Boxplots show the median, 1st and 3rd quartiles as boxes, with whiskers representing the 5 and 95% intervals.

Supplementary Figure 4:

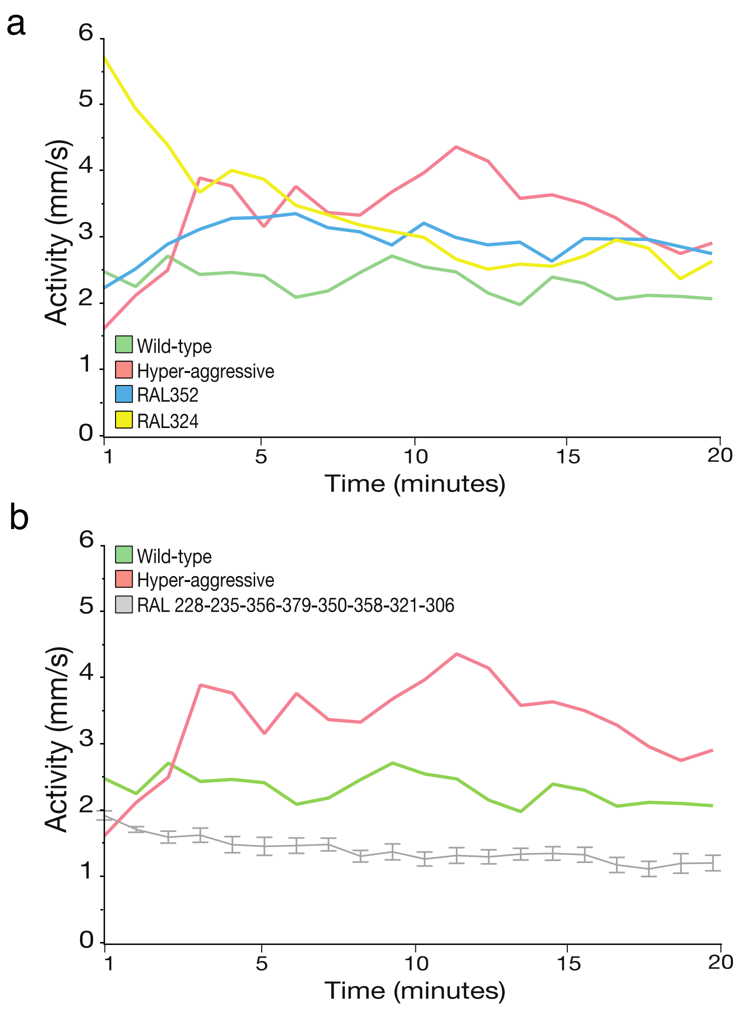

DGRP lines have low activity. a. Activity patterns of the DGRP strains with the highest activity (RAL352 and 324) throughout the 20 min interaction between the two flies are similar to our low aggression control strain (CS) as well as our hyper-aggressive strain. These two DGRP strains represent the strains with the highest (324) and lowest (352) MAS. Their lunge numbers are similar and in the range of the low aggression control strain. **b.** The remaining eight DGRP strains from the group of 10 strains with lowest and highest MAS all exhibit low activity levels. Error bars represent S.E.M.

Supplementary Figure 5:

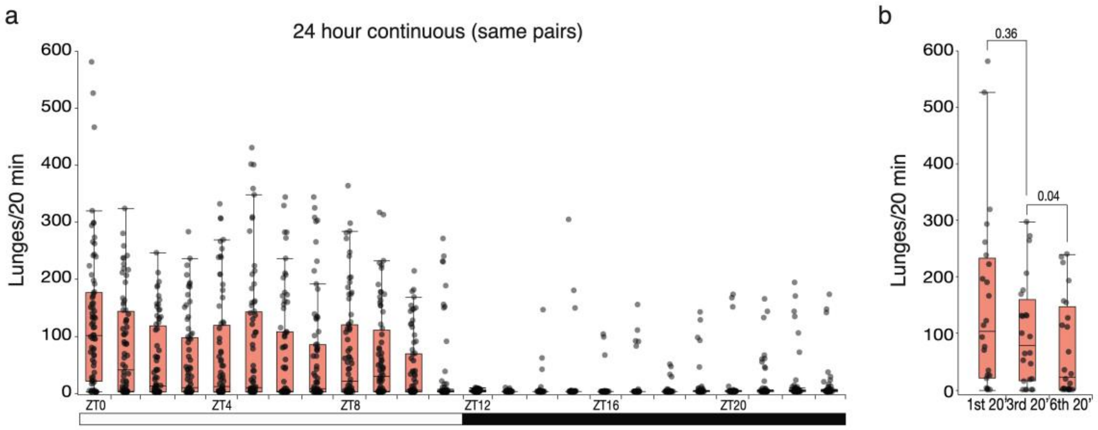

Habituation of aggression over time. a. Continuous recording of 24 pairs of hyper-aggressive flies for 24 hours. The data for each hour is shown as a compilation of each 20 min interval (i.e. each pair is represented 3 times). The median lunge number quickly drops over time. By the evening the median lunge numbers are near zero although a small subset of flies continue to fight throughout the night. **b.** Comparison of lunge numbers of 24 pairs of hyper-aggressive flies in the first 20 min and the last 20 min of the first hour shows a decrease that is not statistically significant (Wilcoxon Rank Sum test, $p = 0.36$). However, by the last 20 min epoch of the 2nd hour, the lunge number median has decreased more than 4 fold, which is statistically significantly lower than in the first 20 min (Wilcoxon Rank Sum test, $p = 0.04$, n =24 pairs). Boxplots show the median, 1st and 3rd quartiles as boxes, with whiskers representing the 5 and 95% intervals. Boxplots show the median, 1st and 3rd quartiles as boxes, with whiskers representing the 5 and 95% intervals.

Supplementary Figure 6:

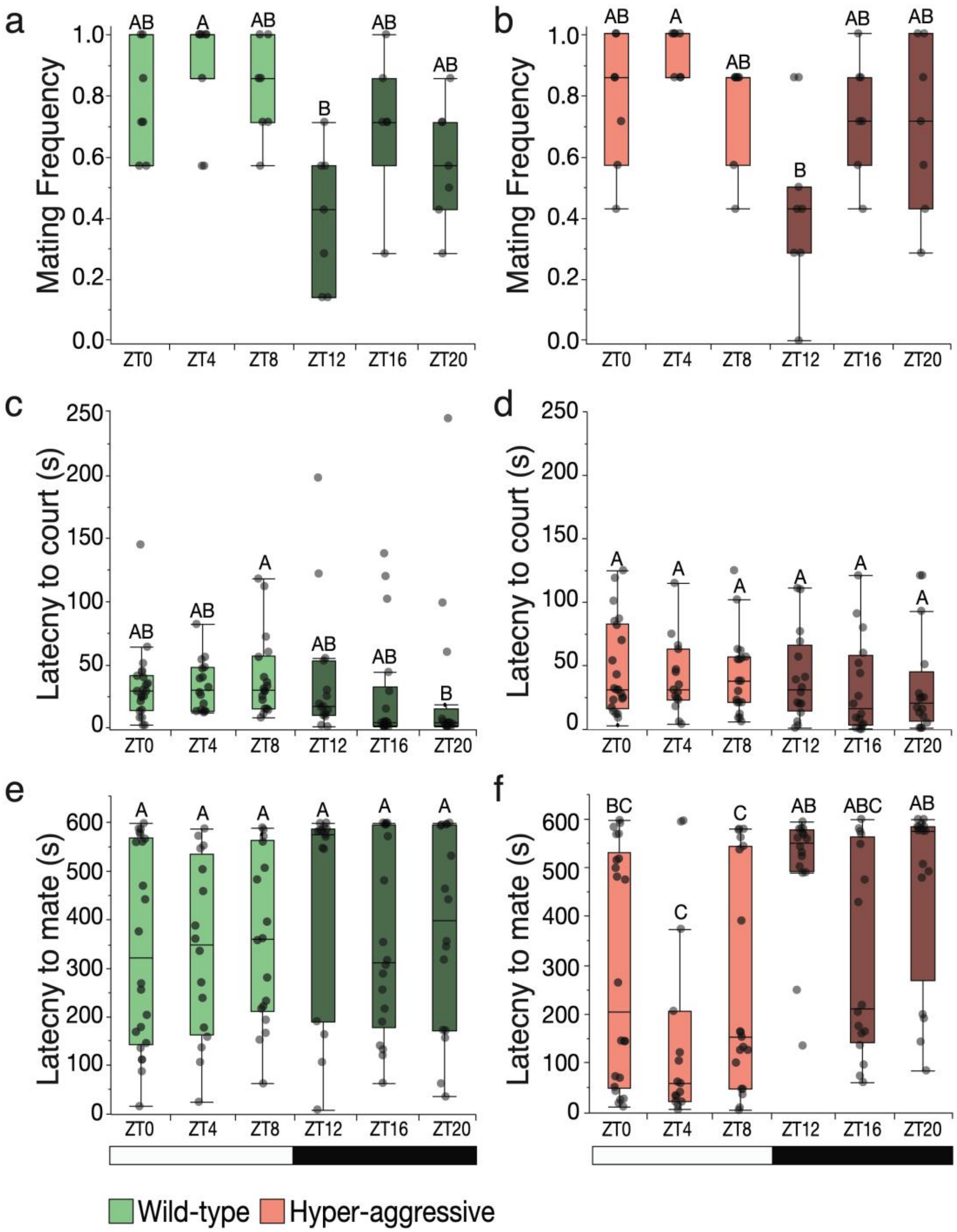

24h variation of mating frequency, and of courtship and mating latency. a. Mating frequency variation over the different times of the day in the low aggression control strain. During the day timepoints, mating frequency is very high, the frequency

significantly decreases at lights off and then increases again during the night (Kruskal-Wallis ANOVA with Dunn's test and Bonferroni correction, significant differences are denoted with letters, ZT12 vs. ZT4, $p = 0.0028$, n = 7 replicates of 6-7 pairs each). **b.** A similar pattern is observed in the hyper-aggressive strain (Kruskal-Wallis ANOVA with Dunn's test and Bonferroni correction, ZT12 vs. ZT4, $p = 0.002$, n = 7 replicates of 6-7 pairs each). **c-d.** While we observed daily variations in the courtship index, we observed no clear pattern in the latency to court in either strain. Only one timepoint comparison reached statistical significance in the low aggression control strain (Kruskal-Wallis ANOVA with Dunn's test and Bonferroni correction, significant differences are denoted with letters, $CS^{ZT20\text{ vs. }ZT8}$, $p = 0.019$, n = 15-22 pairs per timepoint) **e.** Although the latency to mate was longest at the beginning of the night, none reached statistical significance across all timepoints for the low aggression control strain. **f.** In the hyper-aggressive strain, latencies were also longest at the beginning of the night. However, in this strain the latencies were markedly shorted during the day-time than at night (Kruskal Wallis ANOVA with Dunn's test and Bonferroni correction, significant differences are denoted with letters, $Aggr^{ZT20\text{ vs. }ZT4}$, $p = 0.0013$; $Aggr^{ZT12\text{ vs. }ZT4}$, $p = 0.0025$; $Aggr^{ZT20\text{ vs. }ZT8}$, $p = 0.056$, n = 15-22 pairs per timepoint). Boxplots show the median, 1st and 3rd quartiles as boxes, with whiskers representing the 5 and 95% intervals. Boxplots show the median, 1st and 3rd quartiles as boxes, with whiskers representing the 5 and 95% intervals.

<u>Supplementary Video 1</u>: Assembly of the Divider Assay. Video illustrating the assembly of the Divider Assay. A transparent food source is made with an agar base food that contains sugar and corn syrup. The chamber is laid down on the food with inserted opaque dividers. A glass plate covers the chamber. Flies are loaded a few hours after they eclose by CO_2 anesthesia and gently dropped on either side of the dividers with a paint brush. The glass cover plate is moved over the arenas as the flies are loaded from the back to the front of the chamber. Flies are then stored in the isolated state in the chambers in a light controlled room until they are tested for aggression. On the day of the recording, the assembled set up with flies is gently placed onto an LED light pad to illuminate the flies from below (shown in the image above) except in experiments performed in the dark where an infrared light source is used. Dividers are gently removed, the flies are video taped for 20 min and the video recording is analyzed using the automated JAABA classifier.

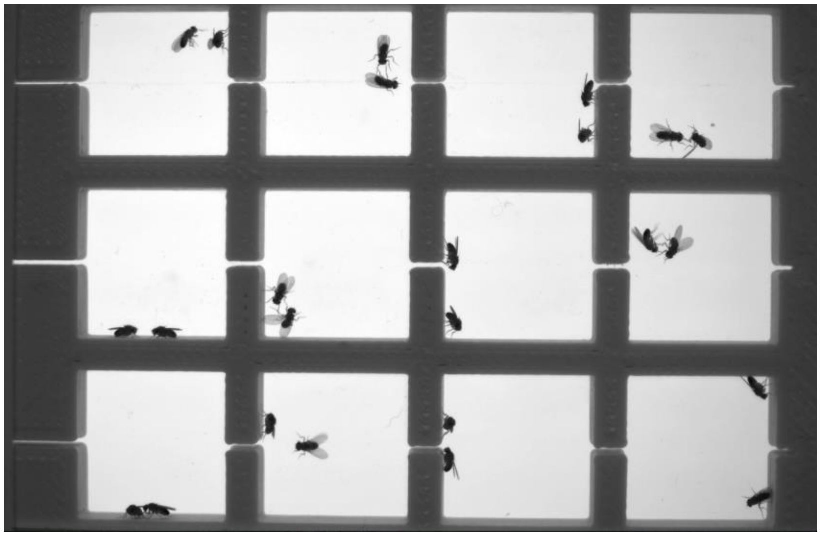

<u>Supplementary Video 2</u>: Raw lunging and boxing, tracking and classifier performance. a. Lunging and high-level boxing (reciprocal lunging) in real time (real time speed 20 FPS) in a pair of flies in one square arena in the Divider Assay. **b.**

Tracking consistency during lunging and boxing using FlyTracker. Video is slowed down to better illustrate behavior. **c-d.** Prediction of lunging using JAABA by our trained "Lunge" classifier (Bottom panel in blue; single frame with lunges indicated by a vertical red stipe) for the dyadic pair (Orange and Green). Accuracy of classifier is observable in reciprocal lunging where fly IDs remain separate and behavior is correctly assigned to the specific fly engaged in lunging. Marking of single frames and accuracy of classification reduces erroneous counting of lunges. Video playback speed is at 1/10th of real time.

Supplementary .stl files:

3D-print files of the different Divider Assay Chambers. Tinkercad.stl files with the Divider Assay standard chamber design. Two variant .stl files with chambers with increased surface area are also shown (5x and 13x surface area). Each design also includes 4 or 3 standard arenas to run parallel to the larger arenas. The .stl files can be opened and modified with any design software.
