# Supplementary material for: The Divider Assay is a high-throughput pipeline for aggression analysis in Drosophila
Source: Commun Biol. 2021 Jan 19;4:85. doi: 10.1038/s42003-020-01617-6 (PMC7815768; doi:10.1038/s42003-020-01617-6)
Supplement: Supplementary file 3 — Description of Supplementary Files [file 42003_2020_1617_MOESM3_ESM.pdf]

Description of Additional Supplementary Files

File name: Supplementary Video 1

Description: Assembly of the Divider Assay. Video illustrating the assembly of the Divider Assay. A transparent food source is made with an agar base food that contains sugar and corn syrup. The chamber is laid down on the food with inserted opaque dividers. A glass plate covers the chamber. Flies are loaded a few hours after they eclose by CO2 anesthesia and gently dropped on either side of the dividers with a paint brush. The glass cover plate is moved over the arenas as the flies are loaded from the back to the front of the chamber. Flies are then stored in the isolated state in the chambers in a light controlled room until they are tested for aggression. On the day of the recording, the assembled set up with flies is gently placed onto an LED light pad to illuminate the flies from below (shown in the image above) except in experiments performed in the dark where an infrared light source is used. Dividers are gently removed, the flies are video taped for 20 min and the video recording is analyzed using the automated JAABA classifier.

File name: Supplementary Video 2

Description: Raw lunging and boxing, tracking and classifier performance. a. Lunging and high-level boxing (reciprocal lunging) in real time (real time speed 20 FPS) in a pair of flies in one square arena in the Divider Assay. b. Tracking consistency during lunging and boxing using FlyTracker. Video is slowed down to better illustrate behavior. c-d. Prediction of lunging using JAABA by our trained “Lunge” classifier (Bottom panel in blue; single frame with lunges indicated by a vertical red stipe) for the dyadic pair (Orange and Green). Accuracy of classifier is observable in reciprocal lunging where fly IDs remain separate and behavior is Herman A. Dierick, M.D. Associate Professor Dept Mol Hum Genetics One Baylor Plaza, Room S940 Houston, Texas 77030 e-mail: dierick@bcm.edu Tel: 713-798-6865 correctly assigned to the specific fly engaged in lunging. Marking of single frames and accuracy of classification reduces erroneous counting of lunges. Video playback speed is at 1/10th of real time.

File name: Supplementary Data 1

Description: Raw data corresponding to the main figures in the manuscript. The Excel sheet contains sheet tabs for each figure showing the raw data points for each experiment corresponding to the replicates indicated in each figure in the manuscript.

File name: Supplementary Data 2

Description: 3D-print files of the standard Divider Assay Chamber. Tinkercad.stl file with the Divider Assay standard chamber design of 4x3 arenas. The .stl files can be opened and modified with any design software.

File name: Supplementary Data 3

Description: 3D-print files of two variant Divider Assay Chambers. Tinkercad.stl files for chambers with increased surface area: 5x and 13x surface area. Each design also includes 4 (for the 5x design) or 3 (for the 13x design) standard arenas to run parallel to the larger arenas. The .stl files can be opened and modified with any design software.
