# Supplementary material for: The Divider Assay is a high-throughput pipeline for aggression analysis in Drosophila
Source: Commun Biol. 2021 Jan 19;4:85. doi: 10.1038/s42003-020-01617-6 (PMC7815768; doi:10.1038/s42003-020-01617-6)
Supplement: Supplementary file 9 — Reporting Summary [file 42003_2020_1617_MOESM9_ESM.pdf]

nature research

Corresponding author(s): Herman Dierick

Last updated by author(s): Oct 25, 2020

Reporting Summary

Nature Research wishes to improve the reproducibility of the work that we publish. This form provides structure for consistency and transparency in reporting. For further information on Nature Research policies, see our Editorial Policies and the Editorial Policy Checklist.

Statistics

For all statistical analyses, confirm that the following items are present in the figure legend, table legend, main text, or Methods section.

n/a	Confirmed	
☐	☒	The exact sample size (*n*) for each experimental group/condition, given as a discrete number and unit of measurement
☐	☒	A statement on whether measurements were taken from distinct samples or whether the same sample was measured repeatedly
☐	☒	The statistical test(s) used AND whether they are one- or two-sided<br>*Only common tests should be described solely by name; describe more complex techniques in the Methods section.*
☐	☒	A description of all covariates tested
☐	☒	A description of any assumptions or corrections, such as tests of normality and adjustment for multiple comparisons
☐	☒	A full description of the statistical parameters including central tendency (e.g. means) or other basic estimates (e.g. regression coefficient) AND variation (e.g. standard deviation) or associated estimates of uncertainty (e.g. confidence intervals)
☐	☒	For null hypothesis testing, the test statistic (e.g. *F*, *t*, *r*) with confidence intervals, effect sizes, degrees of freedom and *P* value noted<br>*Give P values as exact values whenever suitable.*
☒	☐	For Bayesian analysis, information on the choice of priors and Markov chain Monte Carlo settings
☒	☐	For hierarchical and complex designs, identification of the appropriate level for tests and full reporting of outcomes
☐	☒	Estimates of effect sizes (e.g. Cohen's *d*, Pearson's *r*), indicating how they were calculated

Our web collection on statistics for biologists contains articles on many of the points above.

Software and code

Policy information about availability of computer code

Data collection	JAABA was used for automated video analysis with a classifier developed and tested here within the platform using machinelearning
Data analysis	Data was statistically analyzed with Prism GraphPad version 8.4.1

For manuscripts utilizing custom algorithms or software that are central to the research but not yet described in published literature, software must be made available to editors and reviewers. We strongly encourage code deposition in a community repository (e.g. GitHub). See the Nature Research guidelines for submitting code & software for further information.

Data

Policy information about availability of data

All manuscripts must include a data availability statement. This statement should provide the following information, where applicable:

- Accession codes, unique identifiers, or web links for publicly available datasets
- A list of figures that have associated raw data
- A description of any restrictions on data availability

All raw data and video files are available from the authors upon request

Field-specific reporting

Life sciences study design

All studies must disclose on these points even when the disclosure is negative.

Sample size	Sample size was determined on a test set with different levels of aggression and effect sizes were determined to generate sample numbers.
Data exclusions	No data were excluded
Replication	Many replicates are included in the manuscript showing high reproducibility between replicates. All these data are shown in the manuscript with sample numbers and statistical analysis.
Randomization	Random animals were collected from each genotype.
Blinding	Data were analyzed mostly by automated video analysis so a person was not involved in scoring. For experiments with manual observers, 3 observers were used and 2 of these were blind to the data. No statistical differences were found between observer scores and the scores were not significantly different from the automated scoring system.

Reporting for specific materials, systems and methods

We require information from authors about some types of materials, experimental systems and methods used in many studies. Here, indicate whether each material, system or method listed is relevant to your study. If you are not sure if a list item applies to your research, read the appropriate section before selecting a response.

Materials & experimental systems

n/a	Involved in the study	
☒	☐	Antibodies
☒	☐	Eukaryotic cell lines
☒	☐	Palaeontology and archaeology
☐	☒	Animals and other organisms
☒	☐	Human research participants
☒	☐	Clinical data
☒	☐	Dual use research of concern

Methods

n/a	Involved in the study	
☒	☐	ChIP-seq
☒	☐	Flow cytometry
☒	☐	MRI-based neuroimaging

Animals and other organisms

Policy information about studies involving animals; ARRIVE guidelines recommended for reporting animal research

Laboratory animals	We used CantonS and a derived high aggression strain as well as strains obtained from the BDSC listed in the manuscript. All were previously published and references are provided in the relevant sections of the manuscript.
Wild animals	Not applicable.
Field-collected samples	Not applicable.
Ethics oversight	None required for Drosophila.

Note that full information on the approval of the study protocol must also be provided in the manuscript.

April 2020
